# Silicon and Nitrate Differentially Modulate the Symbiotic Performances of Healthy and Virus-Infected *Bradyrhizobium*-nodulated Cowpea (*Vigna unguiculata*), Yardlong Bean (*V. unguiculata* subsp. *sesquipedalis*) and Mung Bean (*V. radiata*)

**DOI:** 10.3390/plants6030040

**Published:** 2017-09-15

**Authors:** Maria Luisa Izaguirre-Mayoral, Miriam Brito, Bikash Baral, Mario José Garrido

**Affiliations:** 1 Instituto Venezolano de Investigaciones Científicas, Centro de Microbiología y Biología Celular, Caracas 1020-A, Venezuela; mlizaguirre@gmail.com; 2 Laboratorio de VirologíaVegetal, Facultad de Agronomía, Universidad Central de Venezuela, Maracay 1050,Venezuela; miriambri@gmail.com (M.B.); garridom@agr.ucv.ve (M.J.G.); 3 Department of Biochemistry, University of Turku, Turku 20500, Finland

**Keywords:** amino acid, *Cowpea chlorotic mottle bromovirus*, *Cowpea mild mottle carlavirus*, root nodulation, ureides

## Abstract

The effects of 2 mM silicon (Si) and 10 mM KNO_3_ (N)—prime signals for plant resistance to pathogens—were analyzed in healthy and *Cowpea chlorotic mottle virus* (CCMV) or *Cowpea mild mottle virus* (CMMV)-infected *Bradyrhizobium*-nodulated cowpea, yardlong bean and mung bean plants. In healthy plants of the three *Vigna* taxa, nodulation and growth were promoted in the order of Si + N > N > Si > controls. In the case of healthy cowpea and yardlong bean, the addition of Si and N decreased ureide and α-amino acids (AA) contents in the nodules and leaves in the order of Si + N> N > Si > controls. On the other hand, the addition of N arrested the deleterious effects of CCMV or CMMV infections on growth and nodulation in the three *Vigna* taxa. However, the addition of Si or Si + N hindered growth and nodulation in the CCMV- or CMMV-infected cowpea and yardlong bean, causing a massive accumulation of ureides in the leaves and nodules. Nevertheless, the AA content in leaves and nodules of CCMV- or CMMV-infected cowpea and yardlong bean was promoted by Si but reduced to minimum by Si + N. These results contrasted to the counteracting effects of Si or Si + N in the CCMV- and CMMV-infected mung bean via enhanced growth, nodulation and levels of ureide and AA in the leaves and nodules. Together, these observations suggest the fertilization with Si + N exclusively in virus-free cowpea and yardlong bean crops. However, Si + N fertilization must be encouraged in virus-endangered mung bean crops to enhance growth, nodulation and N-metabolism. It is noteworthy to see the enhanced nodulation of the three *Vigna* taxa in the presence of 10 mM KNO_3_.

## 1. Introduction

Cowpea (*Vigna unguiculata* (L.) Walp.), yardlong bean (*Vigna unguiculata* subsp. *sesquipedalis* (L.) Verdc.) and mung bean (*Vigna radiata* (L.) Wilczek) are important grain and fodder legume crops grown across tropical and sub-tropical agro-ecological zones worldwide [1]. Cowpea is preferred by the farmers due to the high nutritional quality of the leaves and seeds [2], as well as the plant tolerance to drought [3] and salinity [4]. Yardlong bean is cultivated mainly for its crisp, long tender pods that are consumed both fresh and cooked [5], while mung bean is widely cultivated for its edible iron-protein-rich seeds, antioxidant capacity and tolerance to mild drought [6,7]. These three grain legumes have in common the ability to fix atmospheric N_2_ via an efficient symbiosis with compatible rhizobial strains belonging mainly to the genus *Bradyrhizobium* [8]. Details of the complex processes involving signaling, recognition by both partners and cell division during the early and late events of the symbiosis were recently discussed [9,10,11]. In *Vigna* species, the establishment of the symbiosis is completed with the formation of phaseoloid-type root nodules harboring the rhizobia dedifferentiated into bacteroids and the onset of N_2_ fixation. Inside the nodules, ureides (allantoin and allantoate) are the final products of the symbiotic N_2_ fixation to be exported to the leaves for their catabolism [12]. Concomitantly, the NH_4_^+^ and NH_3_^+^ produced by the ureide catabolism in mature leaves and the NO_3_^−^ reduction in roots are directed mainly toward the synthesis of α-amino acids (AA) as building blocks for protein synthesis and as precursors for hormones and key secondary metabolites that play crucial roles in plant growth and development, including responses to biotic and abiotic stresses [13]. As shown by [14], there is a direct relationship between N availability and the concentrations of AA in different plant organs. In the case of N_2_ fixing grain legumes undergoing systemic virus infections, the effectiveness of the symbiosis is severely hampered by the viruses hijacking the plant cellular machinery for replication [9,15].

*Vigna* species, in general, are highly susceptible to many viruses as described in recent publications [16,17,18,19,20,21,22,23,24,25,26,27,28,29]. Amid *Vigna* infecting virus, the *Cowpea chlorotic mottle* *virus* (family *Bromoviridae*, genus *Bromovirus*)/(CCMV) and the *Cowpea mild mottle virus* (Order Tymovirales, family *Betaflexiviridae*, genus *Carlavirus*)/(CMMV) have emerged in the past two decades as an important threat to legume crops globally [16,29,30,31,32,33,34,35,36,37,38,39]. Unfortunately, cowpea, yardlong bean and mung bean genotypes that are genetically resistant to CCMV or CMMV are not commercially available at present. Therefore, options for controlling these virus infections in field-grown *Vigna* crops are limited to the use of toxic chemicals to restrict the incidence of virus vectors [40,41,42], or of more expensive, and still under experimentation, plant-derived organic chemicals to enhance the systemic acquired resistance without triggering cell death [43]. A plausible alternative to reduce the hindering effects of viruses could be the application of silicon (Si) and NO_3_^−^ (N) to plants. Both Si and N are new emerging prime stress signals for plant resistance to pathogens, influencing the hypersensitive reaction as a component in systemic acquired resistance, via the expression of most pathogen-related genes [44,45,46,47,48].

A number of reports have shown the counteracting effects of Si in virus-infected plants throughout the activation of pathogenesis-related genes [49,50], and as a promoter of nodulation in rhizobia-inoculated cowpea [51], favoring the plant N metabolism [52] and the absorption of nutrients in grain legumes [53,54]. The uptake, transport and benefits of added Si in stressed plants were recently reviewed [55,56]. On the other hand, accumulating evidence had shown the key role played by the reductive conversion of NO_3_^−^/into nitric oxide [57], a signal molecule directly involved in the early events of nodulation [58,59] and in the hypersensitive resistance response to pathogens [60,61,62]. Together, those observations suggest that Si and NO_3_^−^ trigger complex responses to rescue plants from biotic stresses, without collateral negative damage to the symbiotic performance of rhizobia-nodulated plants. Therefore, the aim of this investigation was to determine the effects of Si and NO_3_^-^ on the symbiotic performance of *Bradyrhizobium*-nodulated cowpea, yardlong bean and mung bean plants, either healthy or infected by CCMV or CMMV. The plant analyses included measurements of the nodule mass and aerial dry mass to estimate growth and nodulation, concomitantly with the determination of ureides and AA in the leaves and nodules as indicators of the symbiotic performance of healthy and virus-infected plants subjected to the different plant treatments. 

## 2. Materials and Methods

### 2.1. Plant Materials, Cultural Practices and Experimental Design

Virus-free certified seeds of cowpea, yardlong bean and mung bean were surfaced-sterilized with 70% ethanol for 5 min, rinsed six times with sterile distilled water and pre-inoculated with a *Bradyrhizobium* commercial peat-based inoculant (NovozymesBioAg, Milwaukee, WI, USA), previously proven to be highly efficient on the three *Vigna* taxa. Seeds were immediately sown in sterilized Leonard jars (five seeds per jar) filled with 1 kg of acid-washed sand and 0.8 L of a nutrient solution in the upper and lower compartments, respectively. The basic N-free nutrient solution contained: 2 mM potassium phosphate buffer (pH 5.8), 1 mM MgSO_4_, 2 mM CaCl_2_, 60 μM MnSO_4_, 4 μM H_3_BO_3_, 30 μM Fe-EDTA, 1.6 μM ZnSO_4_, 1.6 μM CuSO_4_ and 0.1 μM NaMoO_4_. To study the effect of NO_3_^−^ (N), the nutrient solution for half of the jars was supplied with 10 mM KNO_3_. This concentration was previously proven in our greenhouse to be physiologically compatible with the *Vigna-Bradyrhizobium* symbiosis. For the Si treatment, half of the plants were grown in N-free or N-supplied nutrient solutions provided with 2 mM silicic acid [63]. Upon germination, 6-days old seedlings were selected for size uniformity and thinned to one plant per jar.

Isolates of CCMV and CMMV were replicated and maintained in cowpea plants. For the virus inoculations, leaves exhibiting the typical CCMV or CMMV symptoms were harvested and macerated in cold 0.1 M phosphate buffer, pH 8.5 (1:2 w:v). The buffered virus extract was rubbed onto the primary leaves of 6-days old plants previously dusted with 600-mesh carborundum. Healthy plants were mock inoculated with phosphate buffer and the abrasive. The inoculated leaves were washed with distilled water to remove any excess inoculum. The plants were grown in an insect proof greenhouse located at map reference coordinates 10°22′ N, 66°58′ W, 1600 m above sea level at the Venezuelan Scientific Research Institute, and were exposed to photosynthetic photon flux densities ≥850 and ≤1100 μmol m^−2^ s^−1^ and average day/night air temperatures of 27 ± 2/20 ± 2 °C. The appropriate nutrient solution was added daily to each Leonard jar to compensate for transpiration losses, and replaced every four days. The plants were spaced up to 40 cm apart to avoid shadowing during growing. Non-Si or N treated healthy plants served as controls.

### 2.2. Yield Parameters

A total of six plant replicates of each treatment were harvested 30-days after germination, at the pre-flowering stage of growth. Plants were then separated into leaves, stems plus petioles, roots and nodules, and the components were individually oven-dried at 80 °C until they reached a constant weight. The combined dry weight of the leaves and stems served as an indicator of growth. The leaf and nodule dried subsamples were extracted in boiling 50 mM phosphate buffer (pH 7.2) and 50% ethanol (v:v). The ureides and α-amino acids (AA) were assayed as described in [64], using a Beckman model 7400 spectrophotometer.

### 2.3. Statistical Analysis

The results were expressed as the statistical mean of combination of six plant replicates per treatment ± the standard error of the mean. The data were statistically analyzed using the one-way analysis of variance based on which LSD values (*p* < 0.05 for *n* = 6) were calculated. The statistical differences between the means were determined using a two-sample F-test for variance followed by a Student´s *t*-test at a *p* ≤ 0.05 level of significance. The analyses were performed with the Sigma Stat 3.1 software. All references to increased or decreased plant parameters as a result of the different treatment combinations were based on values recorded in non-treated healthy control plants.

## 3. Results

### 3.1. Symptoms and Effects of Virus Infection

In cowpea and yardlong bean, the systemic symptoms elicited by CCMV included leaf mottling, yellow mosaic, bright vein clearing and deformations in all trifoliolated leaves (Supplemental Figure S1(1)). However, the symptoms of CMMV infection were mottling, crinkling, puckering, rugosity and a mild mosaic in the leaves. In mung bean, the infections by CCMV or CMMV caused the appearance of a mild mosaic (Supplementary Figure S1(1)). A hypersensitive reaction took place only in the CCMV inoculated primary leaves of mung bean, as evidenced by the appearance of local necrotic lesions (Supplementary Figure S1(2)). In CCMV- or CMMV-infected plants of the three *Vigna* taxa, phaseoloid-type of nodules were formed mainly on the uppermost root whorls, with sparse nodulation in the lower primary and lateral roots. This contrasted with nodulation in healthy plants, where nodules were found uniformly scattered along the root system (Supplemental Figure S2). For all healthy or virus-infected plants, the root dry mass followed the same trend as the aerial mass for each plant-treatment combination (not shown).

### 3.2. Physiology of Healthy Cowpea

**Figure 1:**
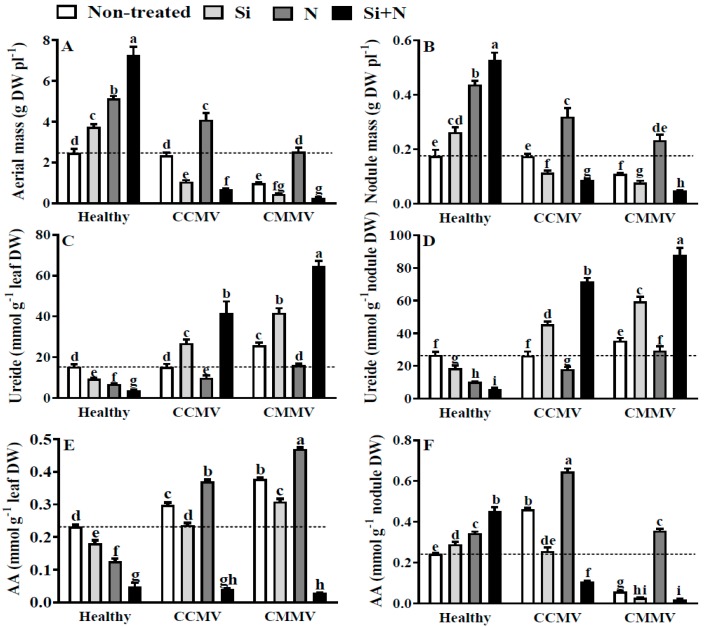
Effects of 2 mM silicic acid (Si), 10 mM KNO_3_ (N) and the combination of Si + N on the aerial mass (**A**), nodule mass (**B**), ureide content in the leaves (**C**) and nodules (**D**), and amino acids (AA) content in the leaves (**E**) and nodules (**F**) of healthy and *Cowpea chlorotic mottle virus* (CCMV) or *Cowpea mild mottle virus* (CMMV)-infected 30-days old cowpea (*V. unguiculata*). Dotted line serves as reference to value of healthy non-treated control. Means of six replicates (±SE) in bars labeled with the same letter(s) are not significantly different (LSD, *p* < 0.05).

The growth of healthy non-treated cowpea was promoted by the addition of Si (1.5 fold), N (2.1 fold) or Si + N (3 fold) (Figure 1A). Nodulation was also enhanced by the addition of Si (1.5 fold), N (2.6 fold) or Si + N (3.2 fold) (Figure 1B). However, the leaf ureide content was reduced by the addition of Si (−1.7 fold), N (−2.4 fold) or Si + N (−4 fold) (Figure 1C). Concomitantly, the ureide content in nodules of healthy plants was reduced by the addition of Si (−1.3 fold), N (−2 fold) or Si + N (−4 fold) (Figure 1D). The leaf AA content was also reduced by the addition of Si (−1.3 fold), N (−1.9 fold) or Si + N (−5 fold) (Figure 1E), with contrast to the increase in the nodule AA content caused by the addition of Si. 

### 3.3. Physiology of CCMV-Infected Cowpea

When compared to controls, and in the absence of Si or N treatments, CCMV infection did not affect the plant growth (Figure 1A) and nodulation (Figure 1B). Visual observations did not reveal an increase in the severity of symptoms in the leaves of Si or Si + N CCMV-infected plants, as compared with non-treated CCMV-infected ones. In contrast, N-treated CCMV-infected plants displayed less severity of virus symptoms. The observed negative effects of Si in CCMV-infected plants were at the level of growth (−2.5 fold) and nodulation (−1.6 fold), in opposition to the increased growth (1.7 fold) and nodulation elicited by the addition of N (1.8 fold). While the combination of Si + N reduced to a minimum the growth and nodulation of CCMV-infected plants, the magnitude of virus symptoms visible in leaves were not augmented by the addition of Si + N. In turn, the leaf and nodule ureide contents were not altered by the CCMV infection in non-treated plants (Figure 1C,D). Yet, the leaf ureide content was increased by the addition of Si (1.8 fold) or Si + N (2.8 fold) and decreased by the addition of N (−1.5 fold). The nodule ureide content was also increased by the addition of Si (1.8 fold) or Si + N (2.9 fold), in sharp contrast to the reduction caused by the addition of N (−1.5 fold). On the other hand, the leaf AA content was increased by 1.3 fold in the CCMV-infected non-treated plants. However, the leaf and nodule AA contents were not altered by the addition of Si, but were increased by the addition of N in leaves (1.6 fold) and nodules (2.6 fold). The combination of Si + N caused significant reduction in AA content of both leaves and nodules.

### 3.4. Physiology of CMMV-Infected Cowpea

When compared to the healthy non-treated controls and in the absence of Si or N treatments, the CCMV infection reduced the plant growth (−2.5 fold) and nodulation (−1.5 fold) (Figure 1A,B). Growth was further reduced by the addition of Si (−5 fold), but increased by the addition of N up to control values. On the other hand, nodulation was reduced by the addition of Si (−2.2 fold). This contrasts with the N promotion of nodulation in CMMV-infected plants that reached control values. Concomitantly, minimum growth and nodulation were observed in CMMV-infected plants supplied with Si + N. The CMMV infection also increased the leaf ureide content (1.6 fold) in non-treated plants (Figure 1C). A further accumulation of ureides in leaves was elicited by the addition of Si (2.8 fold). In contrast, the addition of N restored to control values the leaf ureide content in CMMV-infected ones. In nodules of non-treated plants, the ureide content was increased by 1.4 fold by the CMMV infection (Figure 1D). The addition of Si cause more increment in the nodule ureide content (2.4 fold) contrasting to the effect of added N that restored the nodule ureide content to control values. For all CMMV-infected plants, the addition of Si + N caused an increase in the ureide content in leaves (4.3 fold) and nodules (3.5 fold). On the other hand, the leaf AA content was increased by 1.7 fold in the CMMV-infected non-treated plants (Figure 1E). The leaf AA content was increased by the addition of Si (1.3 fold) or N (2 fold) (Figure 1E). However, the nodule AA content was reduced by the addition of Si (−10 fold) but increased by the addition of N (1.4 fold) (Figure 1F). The combination of Si + N caused reduction of AA content to the minimum level in the leaves and nodules of the CMMV-infected plants. As in the case of CCMV infection, visual observations did not indicate greater severity of virus symptoms in the leaves of Si or Si + N treated CMMV-infected plants. Nonetheless, a marked amelioration of the virus symptoms was observed in N treated CMMV-infected plants.

### 3.5. Physiology of Healthy and Virus-Infected Yardlong Bean

**Figure 2:**
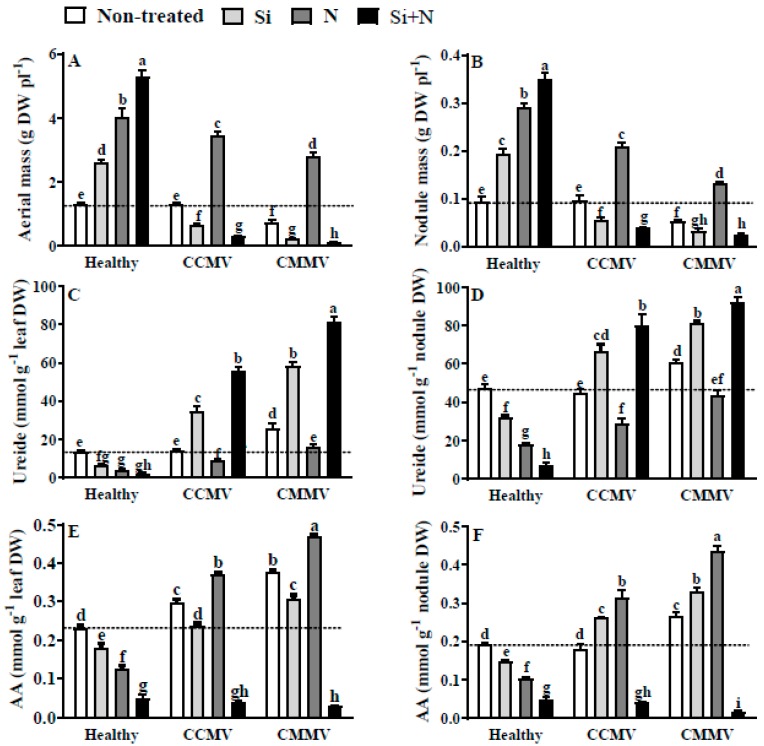
Effects of 2 mM silicic acid (Si), 10 mM KNO_3_ (N) and the combination of Si + N on the aerial mass (**A**), nodule mass (**B**), ureide content in the leaves (**C**) and nodules (**D**), and amino acids (AA) content in the leaves (**E**) and nodules (**F**) of healthy and *Cowpea chlorotic mottle virus* (CCMV) or *Cowpea mild mottle virus* (CMMV)-infected 30-days old yardlong bean (*V. unguiculata* subsp. *sesquipedalis*). Dotted line serves as reference to value of healthy non-treated control. Means of six replicates (±SE) in bars labeled with the same letter(s) are not significantly different (LSD, *p* < 0.05).

Except for the absolute values, the responses of healthy, CCMV- and CMMV-infected yardlong bean plants to the addition of Si, N or Si + N resembled that described for cowpea in terms of aerial mass (Figure 2A), nodule mass (Figure 2B), severity of virus symptoms and ureides content in the leaves and nodules (Figure 2C,D). The only observed difference between cowpea and yardlong bean was the accumulation of AA content in the nodules of CCMV- and CMMV-infected plants (Figure 2E). In yardlong bean, the addition of Si caused respective increment of AA content by 1.4 and 1.7 fold in CCMV- and CMMV-infected plants.

### 3.6. Physiology of Healthy Mungbean

**Figure 3:**
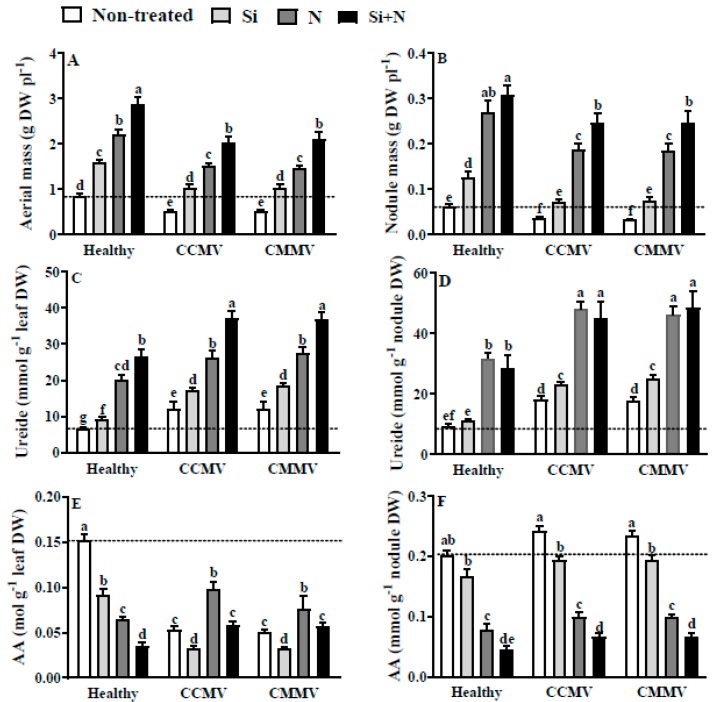
Effects of 2 mM silicic acid (Si), 10 mM KNO_3_ (N) and the combination of Si + N on the aerial mass (**A**), nodule mass (**B**), ureide content in the leaves (**C**) and nodules (**D**), and amino acids (AA) content in the leaves (**E**) and nodules (**F**) of healthy and *Cowpea chlorotic mottle virus* (CCMV) or *Cowpea mild mottle virus* (CMMV)-infected 30-days old mung bean (*V. radiata*). Dotted line serves as reference to value of healthy non-treated control. Means of six replicates (±SE) in bars labeled with the same letter(s) are not significantly different (LSD, *p* < 0.05).

The growth of healthy mung bean was promoted by the addition of Si (2 fold), N (2.8 fold) and Si + N (3.6 fold) (Figure 3A). Nodulation in the controls also positively responded to the addition of Si (2 fold), N (4.2 fold) or Si + N (4.8 fold), with the slightly higher nodulation detected in Si + N plants not reaching statistical significance (Figure 3B). Concomitantly, the leaf ureide content increased by the addition of Si (1.5 fold), N (3 fold) or Si + N (4.2 fold) (Figure 3C). In contrast, the nodule ureides content was not altered by the addition of Si, contrasting with the 3 fold increase in the N or Si + N treated plants (Figure 3D). On the other hand, the AA content in leaves of healthy plants was reduced by the addition of Si (−1.7 fold), N (−2.4 fold) or Si + N (−4.8 fold) (Figure 3E). In parallel, the AA content in nodules of healthy plants was also reduced by Si (−1.3 fold), N (−2.6 fold) or Si + N (−5.3 fold) (Figure 3F).

### 3.7. Physiology of CCMV- or CMMV-Infected Mung Bean

When compared to the healthy non-treated controls, the growth of CCMV- or CMMV-infected non-treated plants was 1.5 fold lower, and there were no significant differences in growth between CCMV- and CMMV-infected plants, regardless of the treatment to which the plants were exposed (Figure 3A). In turn, the addition of Si to CCMV- and CMMV-infected plants increased the growth of the plants up to control values, while the addition of N or Si + N promoted the growth of the virus-infected plants by 2 or 2.7 fold, respectively. Visual observations revealed a marked reduction in the severity of the symptoms in the Si or Si + N virus-infected plants, as compared to the non-treated virus-infected controls. Addition of N almost abolished the symptoms in the virus-infected plants. The nodulation was 2 fold reduced both in CCMV and CMMV infections, and the addition of Si restored nodulation in virus-infected plants to control values (Figure 3B). In the virus-infected plants, the addition of N or Si + N increased nodulation by 2.8 or 3.2 fold, respectively. The CCMV and CMMV infections cause the increment of leaf ureides content by 1.8 fold in the virus-infected non-treated plants (Figure 3C). In turn, the ureides content in leaves of virus-infected plants was further increased by the addition of Si (2.7 fold), N (4.2 fold) or Si + N (5.8 fold). Nevertheless, the nodule ureides content in virus-infected plants was increased by Si (2.4 fold) and by the addition of N or Si + N (4.6 fold) (Figure 3D). The CCMV and CMMV infections similarly reduced the AA content of the leaves (−3.4 fold) (Figure 3E). However, the addition of Si further reduced the amino-acid content of leaves by −4.8 fold, contrasting to the lesser reduction observed in N (−1.6 fold) or Si + N (−2.6 fold) treated virus-infected plants. Infections by CCMV and CMMV did not alter the nodule AA content, regardless of the treatment to which the plants were exposed (Figure 3F).

## 4. Discussion

### 4.1. The Case of Healthy Plants

In the case of healthy *Bradyrhizobium*-inoculated N_2_-fixing cowpea, yardlong bean and mung bean, the augmented growth elicited by Si can be directly attributed to Si for improvement in root nodulation, as previously reported in Si-treated cowpea [51] and *Medicago sativa* cv. Sequel plants [65]. There is also a possibility that the enhanced growth and nodulation in Si treated plants could be as a consequence of the Si promotion of net photosynthesis and chlorophyll content [66], as well as the cytokinin [67], K and Ca levels [63] (the parameters not investigated in current study). Nevertheless, the enhanced growth and nodulation detected in all Si treated healthy plants was far below than that elicited by the combined additions of Si + N. For all healthy plants, this observation seems to be the result of combined synergism of the N added and N from ureides for growth, as well as the enzymatic synthesis of nitric oxide from NO_3_^−^ in the roots and nodules [59,68,69]. In cowpea and yardlong bean plants, the detected inverse relationship between growth and leaf ureide content support the direct participation of Si and N in the promotion of catabolism of ureides in the leaves to generate further N required for the growth. In parallel, the inverse relationship between nodulation and nodule ureide content in the healthy Si, N or Si + N treated cowpea and yardlong bean plants could be also interpreted in terms of a higher ureide export to the aerial organs generated by their greater sink strength linked to greater rates of ureide catabolism in the leaves [12]. In mung bean, however, the direct relationship between growth and leaf ureide content might point to plant metabolic restrictions in the catabolism of ureides resulting in the accumulation of ureides in leaves and the feedback reduction on ureide export from the nodules to the aerial parts. Additionally, the lower AA content in the leaves and nodules of all healthy N or Si + N treated plants also points to an elevated allocation of AA to photosynthesis, thereby increasing N use for CO_2_ fixation and improved nodule efficiency [70]. In the leaves, ureides are catabolized to NH_3_^+^ for its re-assimilation into AA, while NO_3_^−^ in the roots is reduced to AA to be transported in the xylem to the shoot, or can be directly loaded in the xylem to get reduced to AA in the leaves [71,72]. Thus, active export of AA from the leaves to the nodules might have de-repressed the root NO_3_^−^/uptake mechanisms [73], in turn increasing the supply of AA for bacteroid metabolism. In addition, AA synthesized in the leaves and then exported to the nodules may serve as a carbon source for N_2_ fixation in bacteroids [74], triggering the shutdown of NH_3_^+^/assimilation in bacteroids and increasing the synthesis of ureides that are ultimately exported to the aerial organs [12]. The cycling of AA is decisive for maintaining the N balance in mesophyll cells, and the observed reduction of AA and ureides in the leaves and nodules of healthy N or Si + N treated plants suggest the simultaneous use of N-ureide and N-AA for growth. On the other hand, it is known that rhizobia-inoculated legumes grown in the soil with higher NO_3_^−^ content tend to show reduced or nil nodulation and symbiotic N_2_ fixation rates [75]. Nevertheless, the three *Vigna* taxa revealed highest nodulation in the presence of elevated N levels, in agreement with the previous reports [76,77].

### 4.2. The Case of Virus-Infected Plants

The systemic infection of CCMV and CMMV and their hindering effects on the growth and nodulation of cowpea, yardlong bean and mung bean allow the categorization of both viruses as crucial biotic stresses for these three food security key legumes. The typical symptoms of CCMV or CMMV infections were evident in all CCMV- or CMMV-infected N_2_ fixing plants of the three *Vigna* taxa, although, in terms of growth and nodulation, CMMV proved to be more virulent than CCMV in cowpea and yardlong bean plants. It is known that carlaviruses replicate and assemble in the cytoplasm of infected cells [78], with encoded cysteine-rich proteins being the determinants of pathogenicity [79], and the coat proteins being essential for cell-to-cell movement and long-distance transport [80]. In contrast, bromoviruses replicate and assemble in the cytoplasm associated with the endoplasmic reticulum [81,82], and the cell-to-cell movement and long-distance transport of virions are dependent on a non-structural movement protein encoded by the dicistronic genomic RNA3, but independent of the capsid [83]. These differences may account for the enhanced virulence of CMMV in cowpea and yardlong bean, although in mung bean the CCMV and CMMV displayed equal levels of mild virulence. On the other hand, reduced nodulation in virus-infected plants could be the result of virus impairing the early and late events of the symbiosis [9].

In the case of cowpea and yardlong bean plants, the virulence of CCMV and CMMV was further enhanced by Si, causing the greatest reduction in growth and nodulation detected in current investigation. These observations suggest the promotion of antioxidant metabolism by Si rather than Si-triggering the systemic acquired resistance that requires activation of the salicylic acid signaling pathway against virus infections [84]. In contrast, the enhanced nodulation and growth of N treated CCMV- or CMMV-infected plants could be attributed to N compensating for the hindered N_2_ fixation in all virus-infected plants as well as the nitrate availability for the synthesis of nitric oxide required for the activation of the salicylic acid-mediated defense response, and cyanide-resistant respiration pathway, involved in plant resistance to virus infections [85,86,87]. The possibility exists that the negative synergistic effects of Si + N on the growth and nodulation of virus-infected cowpea and yardlong bean plants could be due to the blockage of the antioxidative metabolism and of the brassinosteroids-induced systemic resistance to virus infections allowing the full expression of virus infections [88]. Those suggestions will be the subject of future investigations. Concomitantly, the accumulation of ureides in the leaves of Si or Si + N treated CCMV- and CMMV-infected plants could be ascribed to an impairment of ureide catabolism caused by severe cell ultrastructural damage previously reported in carlavirus-infected [89,90,91] and CCMV-infected plants [92]. Since there are no available reports on ultrastructural alterations in the nodules of CCMV- or CMMV-infected plants, the accumulation of ureides in virus-infected plants must be ascribed to a combination of feedback inhibition and low sink strength of the aerial mass. Similarly, low rates of AA catabolism for growth, lower rates of AA export to the nodules and a lower nodule activity as a result of virus infections could also be factors underlying the accumulation of AA in the leaves and nodules of Si or N treated CCMV- and CMMV-infected plants. This finding agreed with previous reports on the high concentrations of AA in plants infected by compatible viruses [93]. The similarities between cowpea and yardlong bean in terms of growth, nodulation and responses to different treatments can be explained by their close taxonomic relationship (cluster A) [94].

In contrast to cowpea and yardlong bean, the exogenous addition of Si promoted growth and nodulation of CCMV- and CMMV-infected mung bean, with the combination of Si + N triggering maximum growth and nodulation in CCMV- and CMMV-infected plants. In the case of mung bean, addition of Si seemed to activate the systemic acquired resistance to reduce the negative impacts of CCMV and CMMV infections. The amelioration of the virus symptoms visible in the leaves of Si and Si + N treated mung bean could be an indicator of the Si-reduced virus titer, as reported in Si-treated *Papaya ring spot virus-infected* cucumber plants [47]. Differences in the responses of mung bean to virus infections with respect to that of cowpea and yardlong bean could be ascribed to the taxonomic allocation of mung bean in cluster B together with *Vigna mungo* and *V. aconitifolia* [94]. 

## 5. Conclusions

The prolific nodulation and better growth of N treated healthy, CCMV- or CMMV-infected plants support the need of N fertilization for *Bradyrhizobium*-inoculated cowpea, yardlong bean and mung bean crops to compensate for the low rates of N_2_-fixation and the hindering effects of CCMV or CMMV infections. However, the intensification of the deleterious effects elicited by CCMV and CMMV in Si treated N_2_-fixing cowpea and yardlong bean question the general promotion of Si as a prime signal for plant resistance to viruses. These observations contrast to the Si-enhanced biochemical resistance to viruses in mung bean. Based on present results, fertilization with Si alone, or in combination with N, should be exclusively recommended for virus-free cowpea and yardlong bean crops. However, Si + N fertilization must be encouraged in virus-endangered bradyrhizobia-inoculated mung bean crops to enhance the plant growth, nodulation and N-metabolism. Nevertheless, field trials are needed in order to implement a wide use of Si and KNO_3_ in virus-infected areas where combinations, doses and fertilization intervals are tested and adjusted to the crop genetic background as well as the Si and N soil contents.

## Supplementary Materials

The following are available online at www.mdpi.com/2223-7747/6/3/40/s1, Figure S1: Symptoms elicited by *Cowpea chlorotic mottle virus* (CCMV) infection in cowpea (1), yardlong bean (2) and mung bean (3). Note in (3), the necrotic lesions in the CCMV-inoculated primary leaves; Symptoms elicited by *Cowpea mild mottle virus* (CMMV) infection in cowpea (4), yardlong bean (5) and mung bean (6), Figure S2: Bradyrhizobia-nodulated roots of 20-days old *Vigna unguiculata*; (A) healthy plants root and (B) CCMV-infected plants root.

## Abbreviations

AA	α-amino acids
CCMV	Cowpea chlorotic mottle bromovirus
CMMV	Cowpea mild mottle carlavirus
Si	silicic acid treatment
N	nitrate treatment
Si + N	simultaneous addition of silicic acid and nitrate
