# Supplementary material for: Silicon and Nitrate Differentially Modulate the Symbiotic Performances of Healthy and Virus-Infected Bradyrhizobium-nodulated Cowpea (Vigna unguiculata), Yardlong Bean (V. unguiculata subsp. sesquipedalis) and Mung Bean (V. radiata)
Source: Plants (Basel). 2017 Sep 15;6(3):40. doi: 10.3390/plants6030040 (PMC5620596; doi:10.3390/plants6030040)
Supplement: Supplementary file 1 [file plants-06-00040-s001.pdf]

Supplementary Materials: Silicon and Nitrate Differentially Modulate the Symbiotic Performances of Healthy and Virus-Infected *Bradyrhizobium*-nodulated Cowpea (*Vigna unguiculata*), Yardlong Bean (*V. unguiculata* subsp. *sesquipedalis*) and Mung Bean (*V. radiata*)

Maria Luisa Izaguirre-Mayoral, Miriam Brito, Bikash Baral and Mario José Garrido

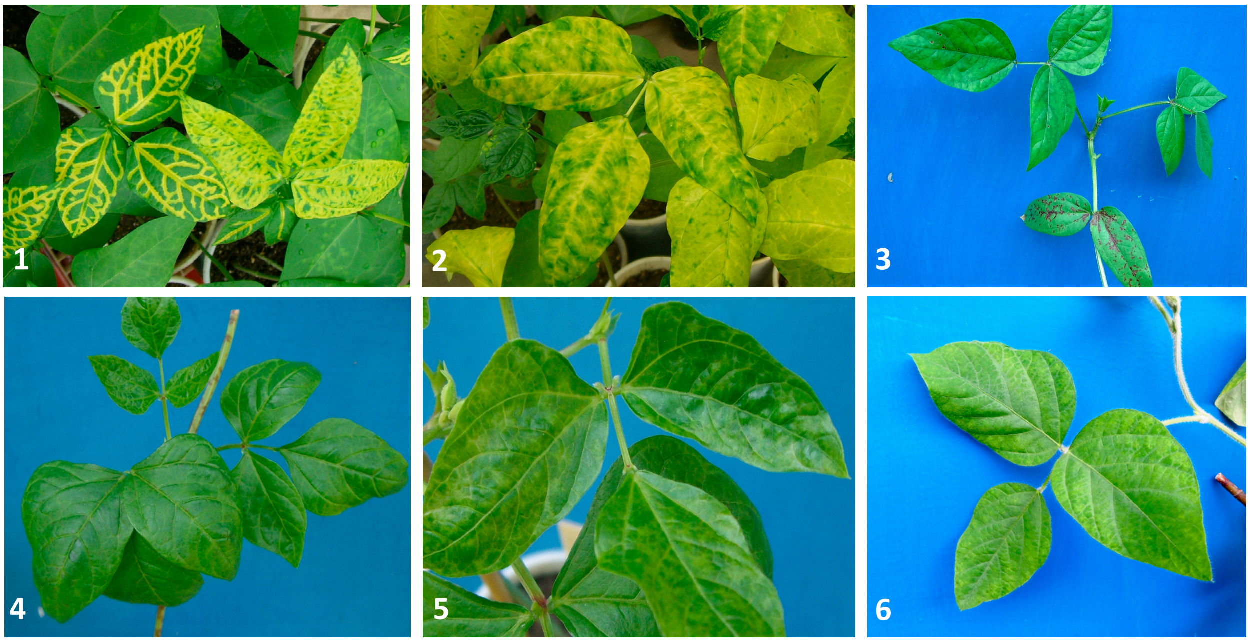

Figure S1. Upper photos: Symptoms elicited by *Cowpea chlorotic mottle virus* (CCMV) infection in cowpea (1), yardlong bean (2) and mung bean (3). Note in (3), the necrotic lesions in the CCMV-inoculated primary leaves. Lower photos: Symptoms elicited by *Cowpea mild mottle virus* (CMMV) infection in cowpea (4), yardlong bean (5) and mung bean (**6**).

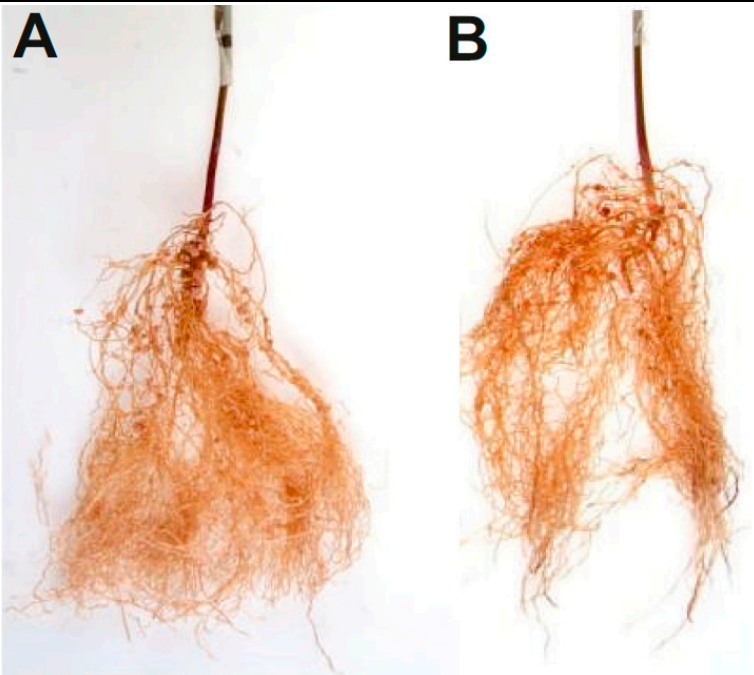

Figure S2. *Bradyrhizobia*-nodulated roots of 20 days old *Vigna unguiculata*; (**A**) healthy plants root and (**B**) CCMV-infected plants root.
